# Analysis of Factors Affecting Quality in Structural Magnetic Resonance Images

**DOI:** 10.1002/hbm.70271

**Published:** 2025-08-01

**Authors:** Lisa Raoul, Anastasia Benedyk, Oksana Berhe, Thomas Leon Kremer, Malika Renz, Yuchen Lin, Niharika Roychoudhury, Alexander Moldavski, Ali Ghadami, Abhijit Sreepada, Marvin Ganz, Markus Sack, Matthias Ruf, Robert Becker, Andreas Meyer‐Lindenberg, Heike Tost, Jamila Andoh

**Affiliations:** ^1^ Department of Psychiatry and Psychotherapy Central Institute of Mental Health, Medical Faculty Mannheim, Heidelberg University Mannheim Germany; ^2^ Department of Neuroimaging Central Institute of Mental Health, Medical Faculty Mannheim, Heidelberg University Mannheim Germany

**Keywords:** CAT12, image quality rating, multi‐scanner, quality control, stability, structural MRI

## Abstract

Combining Magnetic Resonance Images (MRI) from different sources is an increasingly common practice that holds high scientific value. Differences in acquisition parameters and participant characteristics can lead to variations in image quality, highlighting the importance of ensuring these variations do not result in biased statistical outcomes. Here, we investigated contributions of both technical and participant‐related factors to MRI quality. We examined how technical factors (scanner hardware, software, and acquisition protocols) affect the Image Quality Rating (IQR) of anatomical MRI. We also evaluated the stability of IQR over time, examined the effects of defacing on image quality, and investigated how participant characteristics (age, sex, and mental health) influence IQR. We collected 2779 T1‐weighted volumes, acquired at two different scanner sites (both Siemens 3 Tesla), using two coil array designs (64‐channel and 32‐channel array), and four scanner software versions (VB17, VB15, VE11, XA30), five acquisition protocols, including two different spatial resolutions (1 mm, 0.8 mm isotropic). Data were collected from 910 healthy controls (HC) (499 women, mean age 27.55 ± 11.27) and from 563 individuals (321 women, mean age 36.42 ± 12.93) with various clinical conditions (125 Major Depressive Disorder [MDD], 43 Autism Spectrum Disorder [AUT], 81 Alcohol Use Disorder [AUD], 104 Schizophrenia [SZ], 70 Chronic Pain [CP], 41 Bipolar Disorder [BD], and 100 with unspecified disease [NHC]). Structural images were preprocessed and analyzed using the quality control pipelines of the Computational Anatomy Toolbox (CAT12, https://neuro‐jena.github.io/cat12‐help/), which provide an image quality rating (IQR) index for each image, with higher IQR indicating a lower image quality. There was no significant effect of scanner site or coil design on IQR. We found a significant effect of scanner software, with lower image quality for VB17 compared with VB15. There was a significant effect of acquisition protocols (i.e., IQR with protocol “T1_1mm_extended” was higher than with others protocols), and image spatial resolution had a significant impact on IQR, with higher IQR values for 1 mm compared to 0.8 mm. Within participants, IQR was stable across sessions, showing minimal day‐to‐day variability. Defacing had no significant impact on IQR. Regarding participant characteristics, we observed a significant interaction between sex and age: IQR increased with age in men but not in women. Additionally, participants with SZ had a significant higher IQR compared to HC and MDD. This study provides a comprehensive assessment of the influence of technical and participant‐related factors on MRI quality. The findings also support IQR as a robust indicator of image quality and emphasize the importance of integrating image quality metrics, both in multicentric studies and within individual research centers. Incorporating IQR as a quality metric would help minimize biases from image quality variations, enabling a more accurate assessment of underlying structural differences and leading to more reliable findings.

## Graphical abstract

We found that image quality rating (IQR) is influenced by scanner software, MR protocols, spatial resolution, and participant age (increasing in men but not women), while remaining consistent over time. IQR is a reliable metric for improving neuroimaging consistency.

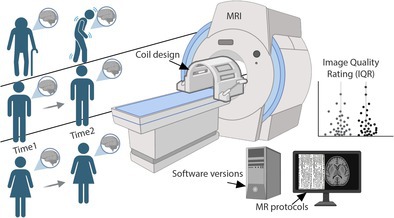

**Summary**

- We assessed the influence of various acquisition and participant‐related parameters on image quality using the Image Quality Rating (IQR) provided by the CAT12 toolbox.
- IQR is influenced by factors such as scanner software, acquisition protocol, and spatial resolution while it remains stable over time. It is also sensitive to participant characteristics, increasing with age in men but not in women.
- These findings highlight the significance of IQR as a robust and reliable quality metric for structural MRI, capable of enhancing consistency and reliability in neuroimaging research, particularly in multi‐center studies.

## 1 Introduction

Pooling magnetic resonance images (MRI) from different projects or research centers has become increasingly important in neuroscience. This approach enables the generation of larger sample sizes, required to develop robust models and improve our understanding of brain‐specific characteristics, particularly in relation to brain disorders (Marek et al. 2022). However, the use of data from different sources, including variations in scanner hardware and software, and from different individuals (sex, age, mental health status), contribute to the creation of heterogeneous datasets, which can introduce biases in the estimation of brain structures and complicate the development of standardized models (Lee et al. 2019; Medawar et al. 2021). A reliable quality index could help to account for variability across different data sources. Such an index would allow for the identification of datasets that meet certain quality thresholds, ensuring that only data with comparable quality metrics are combined.

The influence of scanner upgrades on brain structure estimations has previously been reported (Potvin et al. 2019). The upgrade from Siemens 3 Tesla (3 T) Magnetom Trio to Prismafit platforms (Siemens Medical Systems, Erlangen, Germany), resulting in changes to the main magnet, gradient system, radiofrequency head coil and software updates, resulted in variations in morphometry measures. Differences in acquisition protocols have also been shown to influence cortical measures (Han et al. 2006). In addition, scanners from different manufacturers (Philips, General Electrics) show variability in image quality, with an impact on brain volumes (Wittens et al. 2021). Moreover, a scanner software update led to a fivefold increase in voxel intensity and a 24% increase in the white matter mean signal to noise ratio (SNR), (Shuter et al. 2008).

However, isolating the contributions of hardware, software version, and acquisition protocols is sometimes challenging, as they are often collinear—changes in one are frequently accompanied by changes in others—making it difficult to determine the specific impact of each factor on image quality. These studies are further limited by small sample sizes—three participants with a total of 28 scans in (Potvin et al. 2019), 10 participants with a total of 60 images in (Wittens et al. 2021), and 64 participants with a total of 128 images in (Shuter et al. 2008).

To our knowledge, only one study has examined the impact of acquisition conditions (e.g., scanner hardware, acquisition protocols) on image quality using a large database (Kruggel et al. 2009). In that study, 1073 MRI datasets from 843 subjects were analyzed across 90 scanners and 58 sites, using metrics like SNR, contrast to noise ratio (CNR), mutual information (MI), and white matter/grey matter ratio (WM/GM). Findings showed that acquisition differences significantly affected signal quality; however, the effects of individual parameters, for example, Repetition Time (TR), Echo Time, TE, voxel size, could not be isolated due to collinearity with scanner characteristics.

Furthermore, participant characteristics, for example, age, sex (Ruigrok et al. 2014), and health status (e.g., Harrewijn et al. 2021; Kuo and Pogue‐Geile 2019) are known factors that influence brain structure measurements. For example, brain volume has been reported to be greater in males compared with females, and to change with age (Barnes et al. 2010; Bethlehem et al. 2022; Ruigrok et al. 2014). These effects may be further amplified when data are acquired using different scanners or after scanner updates. For example, the effects of sex on cortical thickness, which were initially significant, disappeared after adjusting for head size, age, and scanner update (Barnes et al. 2010). However, this study did not provide image quality measures that could be used to assess the effects of the scanner update.

Health status is also believed to influence structural measures, though this connection has not been thoroughly explored. A study found that brain volume loss in schizophrenia patients was associated with disease duration (Chand et al. 2020). Such loss in brain volume could impact segmentation algorithms, potentially reducing the accuracy of structural measurements. This suggests that commonly derived structural MRI measures are closely linked to image quality, emphasizing the need for image quality metrics to account for potential variations and ensure the accuracy and reliability of structural analyses.

Several toolboxes have been developed for image quality estimation, for example, CAT12 (https://neuro‐jena.github.io/cat/), FreeSurfer (https://surfer.nmr.mgh.harvard.edu/), MRIQC (https://mriqc.readthedocs.io/en/latest/), many of which provide a variety of metrics that capture unique aspects of noise relevant to structural MRI (Hendriks et al. 2023). This can complicate interpretation, as the metrics often do not converge. Furthermore, implementing these methods becomes particularly challenging when handling large databases. It is therefore essential to establish a composite image quality metric that effectively estimates signal quality and aligns consistently with expert visual quality control assessments.

We used IQR, a validated Image Quality Rating metric that combines image noise, motion‐related bias and resolution implemented in the CAT12 toolbox (Computational Anatomy Toolbox 12) (Gaser et al. 2024) for Statistical Parametric Mapping (https://www.fil.ion.ucl.ac.uk/spm/). This metric was shown to be well‐suited for evaluating image quality in MRI acquired with 3 T Siemens Medical Systems (Bhalerao et al. 2024) and to strongly correlate with human raters' subjective evaluations of quality (Gilmore et al. 2021). Moreover the outcome of IQR was shown to be comparable to the one obtained with other resource and time consuming software packages, for example, FreeSurfer (Singh 2023).

We investigated the effects of various acquisition conditions, including scanner site, coil array types, various scanner software versions, and acquisition protocols including different spatial resolutions, on IQR in 2779 T1‐weighted images. Additionally, we examined the influence of participant characteristics (e.g., age, sex) and medical status (e.g., healthy vs. various mental disorders) on IQR.

The strength of this study lies in its ability to examine the influence of both technical factors and participant characteristics on IQR. This is made possible through the access to a large database, enabling a comprehensive analysis of these factors.

Based on previous literature, we expected scanner site, coil designs, scanner software, and acquisition parameters to have a significant impact on IQR, such that most recent scanner updates and coils with higher channel arrays would have better IQR. Regarding participant characteristics, we expected IQR for patients' data with mental disorders to be worse than for controls because of brain volume loss that has been shown in certain clinical conditions (Chand et al. 2020).

## 2 Methods

Structural images were acquired at the Central Institute of Mental Health in Mannheim, Germany, using a standard sagittal T1‐weighted 3D magnetization‐prepared rapid gradient echo (MPRAGE) sequence. A total of 2779 T1‐weighted anatomical brain scans were acquired with two scanner sites, two coil designs, four scanner software versions, five acquisition protocols, and two image spatial resolutions (1 mm and 0.8 mm isotropic).

### 2.1 Participants

Images were obtained from 1473 individuals categorized by mental health status into a healthy control group and a clinical group. The healthy control group included 910 healthy controls [HC] (499 women, mean age 27.55 ± 11.27), while the clinical group included 563 individuals (321 women, mean age 36.42 ± 12.93; 125 Major Depressive Disorder [MDD], 43 Autism Spectrum Disorder [AUT], 81 Alcohol Use Disorder [AUD], 104 Schizophrenia [SZ], 70 Chronic Pain [CP], 41 Bipolar Disorder [BD], and 100 with unspecified disease [NHC]), Table 1. Eight hundred sixty‐nine participants had two or more assessments.

**TABLE 1:** Sample description.

Mental health status	*n* images	*n* participants	*n* women	Age in years (mean ± SD)
HC	1764	910	501	27.5 ± 11.3
AUD	136	81	29	38.3 ± 12.6
AUT	44	43	20	31.5 ± 9.6
BD	76	41	25	36.3 ± 10.2
CP	138	70	55	49.8 ± 11
MDD	269	125	83	36.3 ± 11.9
SZ	190	104	35	36.4 ± 11.5
NHC	162	99	77	25 ± 7.7

Abbreviations: AUD, Alcohol Use Disorder; AUT, Autism Spectrum Disorder; BD, Bipolar Disorder; CP, Chronic Pain; HC, Healthy control; MDD, Major Depressive Disorder; NHC, non‐healthy control with unspecified disease; SZ, Schizophrenia.

### 2.2 Technical Parameters: Hardware, Software, and Acquisition Parameters

All MR scans were performed on a 3 T Siemens scanner (Medical Systems, Erlangen) at two different sites (namely scanner Heinz and scanner Fritz), both located at the Central Institute of Mental Health (Mannheim, Germany).

Anatomical data were acquired with four different scanner software versions: Tim Trio VB15, Tim Trio VB17, PrismaFit VE11 and PrismaFit XA30. Both scanners received software updates, that is, VB15 (year 2009) and VB17 (2011). Moreover, both scanners received major upgrades including improved gradient system, RF electronics, and head coils: VB17 to VE11 (2018) for scanner Heinz and VB17 to XA30 (2022) for scanner Fritz.

At both scanners, anatomical data were acquired with two coil array designs: 32‐channel and 64‐channel. Both coils have the same structural design, and they differ in the number of coil elements and their placement (i.e., posterior vs. anterior) (Keil et al. 2013).

Five different acquisition protocols were utilized: four of them employed a spatial resolution with a voxel size of 1 mm, while a single acquisition protocol employed a higher spatial resolution of 0.8 mm voxel size. All anatomical data were acquired using a 3D MPRAGE sequence, a FOV 256 × 256 and parallel imaging (GRAPPA) with an acceleration factor of 2. Additional information is provided in Table 2.

**TABLE 2:** Parameters for the five acquisitions protocols: “T1_1mm_short,” “T1_1mm_standard,” “T1_1mm_extended,” “T1_1mm_long,” and “T1_0.8 mm.”

Acquisition protocol	TR/TE (ms)	Number of slices	TI (ms)	Flip angle (°)	Voxel size (mm isotropic)	Bandwidth (Hz/px)	Acquisition time (min)
T1_1mm_short	1570/2.75	176	800	9	1	150	4:30
T1_1mm_standard	2000/3.03	192	900	15	1	130	4:42
T1_1mm_extended	2300/3.03	192	900	9	1	130	5:21
T1_1mm_long	2530/3.8	176	1100	7	1	170	6:03
T1_0.8 mm	2500/3.15	224	1060	8	0.8	130	7:19

Abbreviations: TE, echo time; TI, inversion time; TR, repetition time.

### 2.3 Preprocessing and Image Quality Ratings

We used the CAT12 (Computational Anatomy Toolbox 12, https://neuro‐jena.github.io/cat12‐help/) (Gaser et al. 2024) toolbox integrated within Statistical Parametric Mapping (https://www.fil.ion.ucl.ac.uk/spm/). Preprocessing of anatomical images included spatial denoising, bias and intensity correction, affine registration (using SPM12 tissue probability maps), warping (Geodesic Shooting methods), tissue segmentation and estimation of total intracranial volume (TIV) of each T1‐weighted image. The toolbox also provides a summary score for each image assessing image quality, namely Image Quality Rating (IQR), which is a combination of noise, intensity inhomogeneities, and image resolution. IQR ranges from 0.5 to 10.5, with higher values indicating poorer image quality. We selected CAT12 as our processing tool because it offers an efficient starting point for analysis. It is relatively quick to run (approximately 40–45 min per subject), features an intuitive interface, and operates without requiring extensive computational resources, making it both accessible and practical for large‐scale studies (Gilmore et al. 2021).

### 2.4 Statistical Analysis

All statistical analyses were conducted using R software (version 4.4, The R Foundation for Statistical Computing). Age and TIV were centered and rescaled to allow for standardized comparisons across variables and to facilitate estimated mean prediction.

Some variables were nested within others: the acquisition protocol variable was nested within coil design, the scanner software version variable was nested within the coil design and scanner update variables, and the interaction between scanner software version and acquisition protocol variables was nested within coil design and scanner update as well as scanner site. Scanner software version and acquisition protocol variables were partially crossed (i.e., some scanner software versions were used with only one acquisition protocol, and some acquisition protocols were used with only one scanner software version). For these reasons, we used different models to investigate the effects of specific factors, and we used subsamples of the dataset when appropriate. Model performance was evaluated using r2 function of performance package (Lüdecke et al. 2024). The significance threshold for hypothesis testing was set at *p* < 0.05.

An initial quality check was performed to identify structural images affected by significant motion or other artifacts (e.g., scanner hardware) during data acquisition. For each spatial resolution, using “lme4” (Bates et al. 2014), we fitted a linear mixed model with IQR as the dependent variable, and all our parameters of interest (excluding factors implicated in the nested structure) and TIV as fixed effects. In this model, and in all subsequent models, a random effect was included to account for variability across participants. For each IQR value, we then examined the standardized residuals and Cook's distance. The former identifies outliers and leverage points in the dataset, while the latter helps detect data points with a disproportionate influence on the fitted model. Points with high Cook's distance or with standardized residuals greater than two were considered potential outliers that could affect the stability and interpretation of the model parameters. These criteria are widely used in regression diagnostics to flag influential observations. These images were then subjected to a blind double visual quality check by two independent evaluators (LR and JA). Images that did not pass the visual check were excluded from subsequent analyses (see results section).

*Effect of spatial resolution*: we fitted a linear mixed model with spatial resolution, scanner site, scanner software, TIV, and participant characteristics (health status, age, sex, and their interaction) as fixed effects. The significance of the resolution effect was assessed using Wald F‐tests with the Kenward‐Roger approximation for degrees of freedom, as recommended by Singmann and Kellen (2019), via the “lmerTest” package (Kuznetsova et al. 2017). We then estimated the predicted mean for each spatial resolution, averaged over the levels of sex and health status, and age using the “emmeans” package (Lenth et al. 2024).

After testing for the effect of resolution, we conducted separate analyses for images with 1 mm and 0.8 mm resolution. Images of 0.8 mm resolution were obtained with the same scanner site, scanner software version (VE11), coil design (64 channels), and acquisition protocol (“T1_0.8 mm”).

*Effect of scanner site, coil design, scanner software version and acquisition protocol*: for images at 1 mm spatial resolution, we investigated whether scanner site, coil design, scanner software version, and acquisition protocol significantly predicted image quality. Due to the nested structure of some effects, we fitted a first model with scanner software version, acquisition protocol, TIV, and participant parameters (health status, age, sex, and their interaction) as fixed effects, and with participants as a random effect. The statistical significance of scanner software version and acquisition protocol was evaluated using Wald F‐tests. As both effects were partially crossed, we created a combined variable representing the combination between scanner software version and protocol. We estimated the marginal means for each combination. We defined six specific contrasts aiming to test for differences in IQR in images acquired using different acquisition protocols and six contrasts to test for differences in IQR in images acquired with different scanner software versions. P values were corrected for six multiple comparisons with Sidak method. We fitted a second model with scanner site, coil design, TIV, and participant parameters (health status, age, sex, and their interaction) as fixed effects, and with participants as a random effect. The statistical significance of scanner site and coil design effect were evaluated using Wald F‐tests. We then estimated the marginal means for each level of significant factor and tested for significant differences between them using Wald's T‐test. We also checked for the effect of scanner site and coil design in subsample to account for the nested structure of the factors.

*Stability of IQR over time*: for participants who underwent two anatomical images taken at different time points (T1, T2) using identical acquisition, we assessed whether the IQR at T1 predicted the IQR at T2. We fitted a linear regression model with the IQR at T2 as the dependent variable and the IQR at T1 as the predictor. We included a variable “(time elapsed between T1 and T2) × sex” to account for age × sex interaction effects found in previous analyses. The main effect of the IQR at T1 was assessed using Wald F‐tests (Kenward‐Roger method).

*Defacing*: a widely used procedure to ensure participant confidentiality, involves removing identifiable facial features from the anatomical data, which may influence specific statistical and imaging outcome (Rubbert et al. 2022). We utilized MiDeFace v. 7.4.0 8 (https://surfer.nmr.mgh.harvard.edu/fswiki/MiDeFace), a tool implemented in FreeSurfer designed for the minimally invasive defacing of MRI images, while ensuring privacy protection. To evaluate its potential impact on IQR, we performed a paired Wilcoxon test on a subsample of 190 images, to determine any significant differences between pre‐ and post‐defaced images.

*Effect of participants characteristics*: for images at each spatial resolution (i.e., 0.8 or 1 mm), we examined whether health status, age, sex, and their interaction, predicted IQR. We fitted a mixed model with health status, age, sex, their interaction, TIV, and, for 1 mm the parameters found to significantly predict IQR as fixed effects. The significance of the main and interaction effects was assessed using Wald F‐tests (Kenward‐Roger method). For each main effect not involved in an interaction, we conducted pairwise comparisons by estimating the means for each level and tested for significant differences between them using Wald's T‐test, with a significance threshold adjusted for multiple comparisons using the Tukey–Kramer method. For the interaction between age and sex, we estimated the effect of age on IQR separately for women and men using the emtrends function of “emmeans” package.

## 3 Results

### 3.1 Outliers Detection and Exclusion

Based on Cook's distance and standardized residuals, we identified 51 images (22 women, mean age = 37.73) as potential outliers for the 1 mm resolution. These images were then subjected to visual inspection by two independent evaluators (LR, JA). After visual inspection, 19 images (7 women, mean age = 32.11) were removed because of blurriness due to strong motion artifacts.

For the 0.8 mm resolution, 19 images (10 women, mean age = 46.53) were identified as potential outliers. After visual inspection, 8 images (5 women, mean age = 43.88) were removed. Inter‐rater reliability was 95.71%.

Therefore, 2497 images with a spatial resolution of 1 mm and 255 images with a spatial resolution 0.8 mm (2752 images in total) were used for subsequent analyses.

### 3.2 Effect of Spatial Resolution

**FIGURE 1:**
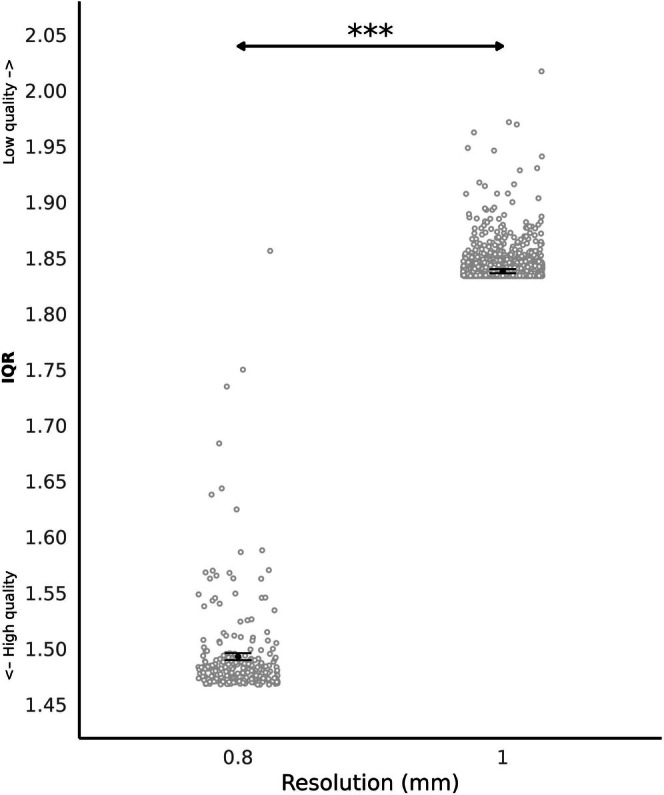
IQR according to spatial resolution (mm). Each grey circle represents an individual IQR value. Black points and lines indicate the estimated marginal means and confidence intervals for each spatial resolution (left: 0.8 mm, right: 1 mm), averaged over the levels of sex and health status. F‐test showed significant difference with *p* < 0.001 (***).

F‐test showed that spatial resolution (nested within protocol) significantly predicted IQR (F(1, 1518) = 64319.31.74, *p* < 0.001) with IQR being higher for anatomical data acquired with 1 mm isotropic (estimated mean = 1.838, 95% CI = [1.837, 1.840]) compared with 0.8 mm isotropic (estimated mean = 1.493, 95% CI = [1.490, 1.496]) (Figure 1).

Using a subsample of images (*n* = 687) acquired with the same coil design (64 channels), scanner site, and scanner software version (VE11) we found that spatial resolution (nested within protocol) significantly predicted IQR (F(1, 386) = 23293.59.72, *p* < 0.001) with IQR being higher for anatomical data acquired with 1 mm isotropic (estimated mean = 1.837, 95% CI = [1.831, 1.843]) compared with 0.8 mm isotropic (estimated mean = 1.492, 95% CI = [1.486, 1.498]) (F‐test conducted with spatial resolution, TIV and participants characteristics as fixed effect).

### 3.3 Influence of Scanner Site, Coil Design, Software Version, and Acquisition Protocol on IQR for Images at 1 mm Resolution

#### 3.3.1 Influence of Scanner Software Version and Acquisition Protocol

F‐tests revealed significant effects of scanner software version (F(3, 2163) = 15.64, *p* < 0.001) and acquisition protocol (F(2, 2198) = 12.63, *p* < 0.001). Estimated marginal means for each combination of scanner software version × acquisition protocol (6 combinations) are reported in Table 3.

**TABLE 3:** Estimated mean for each combination scanner software version × acquisition protocol (nested in scanner update, scanner site and coil design) averaged over the levels of sex and health status.

Scanner site	Coil design	Combination software version × acquisition protocol	Estimated mean	SE	Confidence limits	df
Fritz	32	VB15 × T1_1mm_short	1. 837	0. 001	1.835, 1.839	2147
Fritz	32	VB17 × T1_1mm_extended	1.843	0.001	1.842, 1.844	2176
Heinz	32	VB17 × T1_1mm_long	1.838	0.002	1.835, 1.840	2427
Fritz	32	VB17 × T1_1mm_short	1.840	0.001	1.839, 1.842	2446
Heinz	64	VE11 × T1_1mm_standard	1.838	0.001	1.836, 1.839	2352
Fritz	64	XA30 × T1_1mm_standard	1.840	0.001	1.838, 1.841	1743

We defined contrasts to compare the estimated means across specific levels of the scanner software version × acquisition protocol combination. Six contrasts were designed to test for differences in IQR in images acquired using different acquisition protocols (see Table 4). Another six contrasts were defined to test for differences in IQR in images acquired with different scanner software versions (see Table 5).

**TABLE 4:** Contrasts on estimated mean of scanner software version × acquisition protocol combination that allow to test for differences in IQR in images acquired with different acquisition protocols, *p* values Sidak corrected for six comparisons.

IQR contrast on acquisition protocols	Scanner site	Software versions	Coil design	Estimated difference	SE	Confidence limits	df	*t* ratio	*p*
**T1_1mm_extended > T1_1mm_short**	**Fritz**	**VB17**	**32**	**0.003**	**0.001**	**0.001, 0.004**	**2027**	**3.785**	**0.001**
**T1_1mm_extended > T1_1mm_long**	**Fritz, Heinz**	**VB17**	**32**	**0.005**	**0.001**	**0.001, 0.008**	**2366**	**3.643**	**0.002**
**T1_1mm_extended > T1_1mm_standard**	**Fritz, Heinz**	**VB17, XA30, VE11**	**32, 64**	**0.004**	**0.001**	**0.002, 0.006**	**2163**	**6.197**	**< 0.001**
T1_1mm_short > T1_1mm_long	Fritz, Heinz	VB17	32	0.003	0.002	−0.001, 0.007	2315	1.68	0.45
T1_1mm_standard > T1_1mm_long	Heinz, Fritz	VE11, XA30, VB17	64, 32	0.001	0.001	−0.003, 0.005	2443	0.658	0.986
T1_1mm_standard > T1_1mm_short	Heinz, Fritz	VE11, XA30, VB17, VB15	64, 32	0.000	0.001	−0.002, 0.002	2192	0.017	1.000

*Note:* Bold values indicate statistical significance *p* < 0.05.

**TABLE 5:** Contrasts on estimated mean of scanner software version × acquisition protocol combination that allow to test for differences in IQR in images acquired with different scanner software versions, *p* values Sidak corrected for six comparisons.

IQR contrasts on scanner software versions	Scanner site	Aquisition protocol	Coil design	Estimated difference	SE	Confidence limits	df	*t* ratio	*p*
**VB17 > VB15**	**Fritz**	T1_1mm_short	**32**	**0.003**	**0.001**	**0, 0.006**	**2405**	**2.818**	**0.029**
XA30 > VE11	Fritz, Heinz	T1_1mm_standard	64	0.002	0.001	−0.001, 0.005	2270	1.734	0.406
XA30 > VB17	Heinz, Fritz	T1_1mm_standard, T1_1mm_long, T1_1mm_short	64, 32	0.001	0.001	−0.003, 0.002	1919	0.687	0.983
XA30 > VB15	Fritz	T1_1mm_standard, T1_1mm_short	64, 32	0.003	0.001	−0.000, 0.006	1966	2.265	0.134
VE11 > VB15	Heinz, Fritz	T1_1mm_standard, T1_1mm_short	64, 32	0.001	0.001	−0.003, 0.004	2336	0.478	0.998
VB17 > VE11	Heinz, Fritz	T1_1mm_long, T1_1mm_short, T1_1mm_standard	32, 64	0.003	0.001	0.000, 0.005	2474	2.549	0.064

*Note:* Bold values indicate statistical significance *p* < 0.05.

**FIGURE 2:**
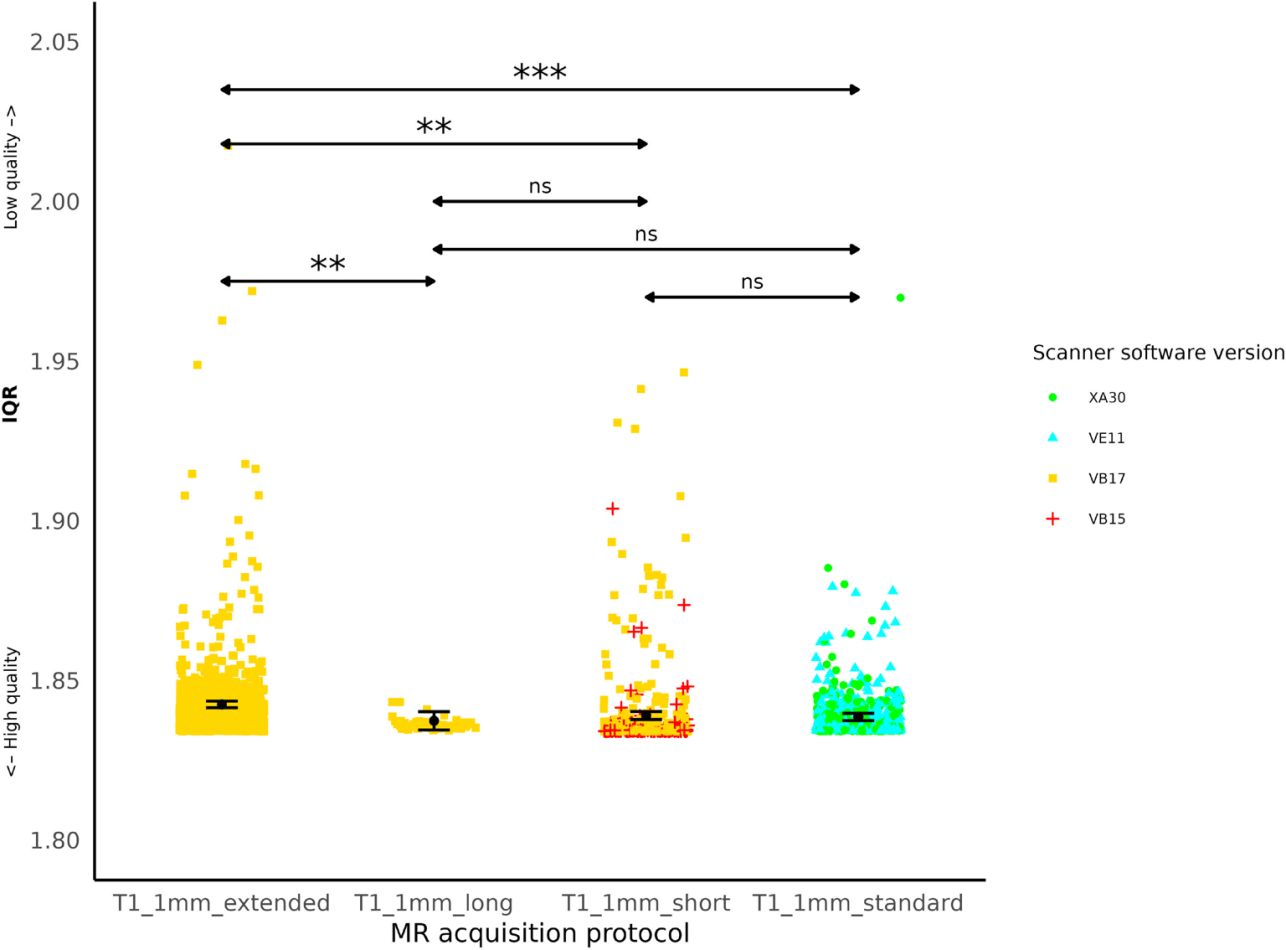
IQR according to acquisition protocol. Each color point represents an individual IQR value. Black points and lines indicate the estimated marginal means and confidence intervals for each acquisition protocol, averaged over the levels of age and health status. Statistically significant differences are shown with *p* < 0.01 (**) and *p* < 0.001 (***), according to T‐tests results.

The contrast “T1_1mm_extended > T1_1mm_short” allowed to test specifically for the effect of acquisition protocol (because images were acquired with the same scanner software version VB17, coil design 32 and scanner site Fritz). It revealed that IQR for protocol “T1_1mm_extended” was significantly higher than IQR for “T1_1mm_short” (beta = 0.003, *p* = 0.001), (see Table 4, line 1 and Figure 2). Two contrasts (Table 4, lines 2 and 4) tested for the effect of acquisition protocol and scanner site (because images were acquired with the same software version VB17 and coil design 32). The contrast “T1_1mm_extended > T1_1mm_long” revealed that IQR for images acquired with “T1_1mm_extended” and in scanner site Fritz was significantly higher than IQR for “T1_1mm_long” in Heinz (beta = 0.005, *p* = 0.002) (see Table 4, line 2 and Figure 2). We then looked at other contrasts to examine the statistical difference between all acquisition protocols. The contrast “T1_1mm_extended > T1_1mm_standard” showed that IQR of images acquired with “T1_1mm_extended,” scanner software version VB17, coil design 32 and in scanner site Fritz was significantly higher than IQR of images acquired with “T1_1mm_standard,” scanner software version XA30 or VE11 and coil design 64 (beta = 0.004, *p* < 0.001) (see Table 4, lines 3–6 and Figure 2). Due to the nested structure of the factors, one could not determine whether the difference can be attributed to the acquisition protocol, the scanner software version or coil design.

**FIGURE 3:**
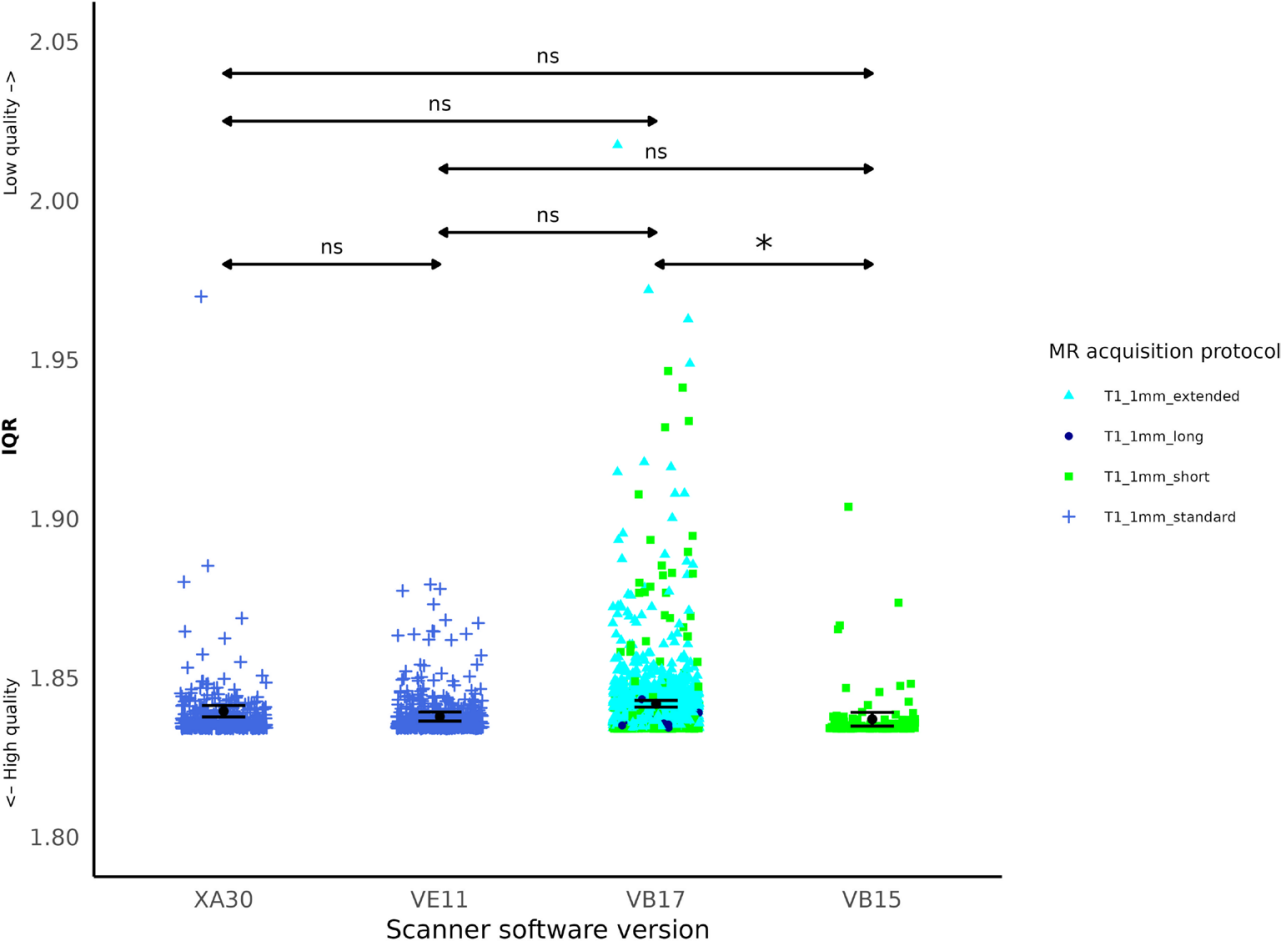
IQR According to scanner software version. Each color point represents an individual IQR value. Black points and lines indicate the estimated marginal means and confidence intervals for each scanner software version, averaged over the levels sex and health status. Statistically significant difference is shown with *p* < 0.05 (*), according to T‐tests results.

IQR for anatomical data acquired with scanner software version VB17 was significantly higher than for scanner software version VB15 (beta = 0.003, *p* = 0.029) (see Table 5, line 1 and Figure 3). The contrast “VB17 > VB15” could specifically examine the effect of scanner software version, as the images were acquired with the same acquisition protocol (“T1_1mm_short”), 32‐channel coil design, and scanner site Fritz (see Table 5, line 1 and Figure 3). There was no significant difference between other contrast on scanner software versions (*p* > 0.1) (see Table 5 lines 2–6). However, due to the nested structure of the factors, one could not determine whether the absence of significant differences could be attributed to the acquisition protocol, the scanner software version, coil design or scanner site.

#### 3.3.2 Influence of Scanner Site and Coil Design

F‐tests on the whole sample revealed significant effects of coil design (F(1, 1714) = 3.87, *p* = 0.049) and scanner site (F(1, 2449) = 10.19, *p* = 0.001).

When we estimated marginal means for each coil on the whole sample averaged over the levels of sex, health status, and scanner site, T‐tests shown that IQR in coil 32 was higher than IQR in coil 64 (beta = 0.001, 95% CI = [0, 0], t(1715) = 1.97, *p* = 0.049). When we estimated marginal means for each scanner site on the whole sample averaged over the levels of sex, health status, and coil design, T‐tests shown that IQR in Fritz was higher than IQR in Heinz (beta = 0.001, 95% CI = [0, 0.002], t(2449) = 3.19, *p* = 0.001). However, T‐test revealed no significant difference between scanner site when we estimated marginal means for each scanner site on subsample of images acquired with scanner software version VB17 and acquisition protocol “T1_1mm_long” or “T1_1mm_short” (beta = 0.003, 95% CI = [−0.00, 0.006], t(2316) = 1.67, *p* = 0.094). Similarly, T‐test reveal no significant difference between scanner site when we estimated marginal means for each scanner site on subsample of images acquired with scanner software version XA30 or VE11 and acquisition protocol “T1_1mm_standard,” (beta = 0.002, 95% CI = [−0.00, 0.004], t(2270) = 1.73, *p* = 0.083). Additionally, when we fitted a model with scanner site and coil design on a subsample of images without images acquired with acquisition protocol “T1_1mm_extended,” the effects of scanner site (F(1, 1252) = 2.63, *p* = 0.10) and coil design (F(1, 1146) = 0.05, *p* = 0.823) become non‐significant. Effects of scanner site and coil design might therefore only be driven by images acquired with acquisition protocol “T1_1mm_extended.”

### 3.4 Stability Over Time

**FIGURE 4:**
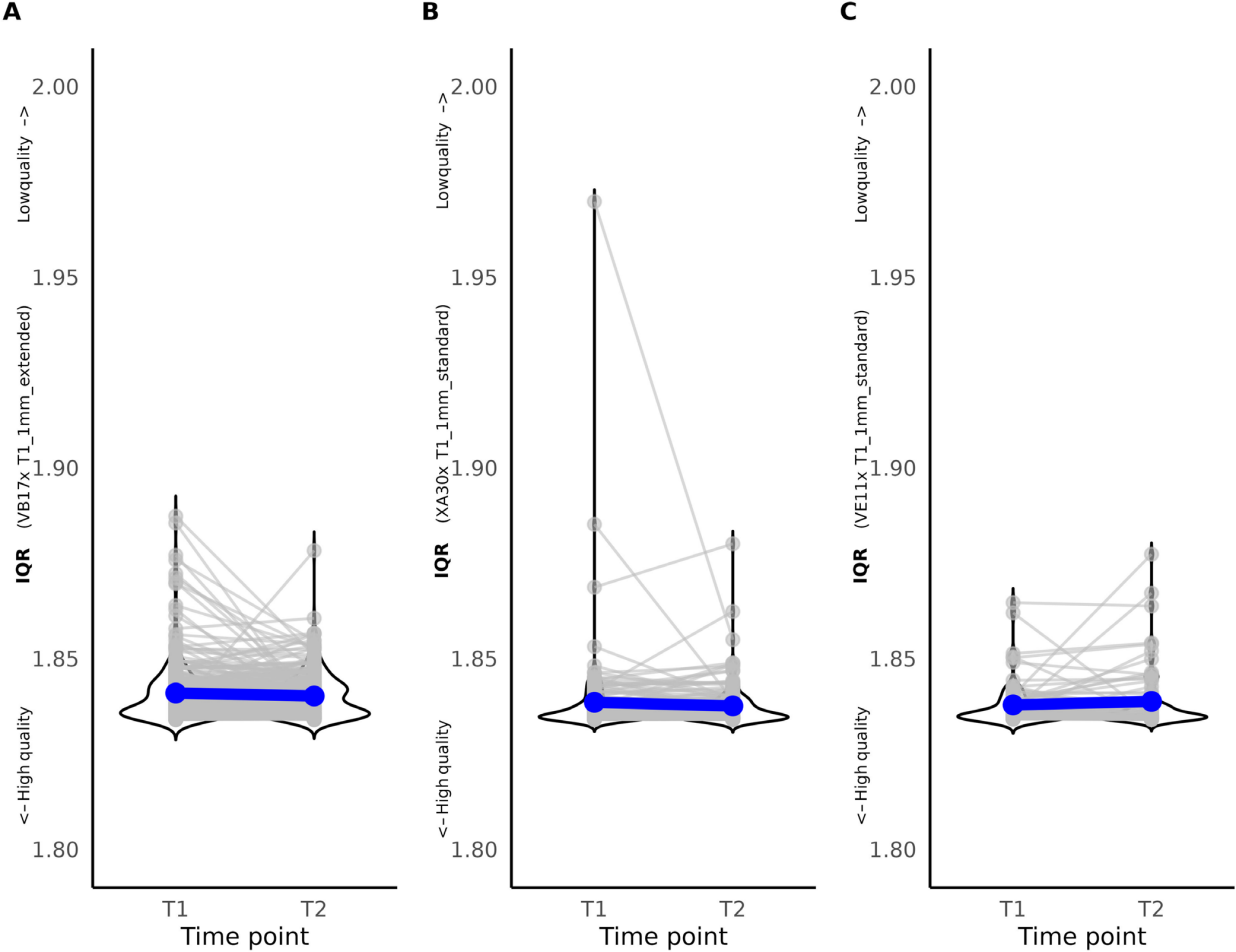
Stability of IQR over time for images acquired with 1 mm spatial resolution. (A) Images acquired with scanner software version VB17 and acquisition protocol “T1_1mm_extended” (*n* = 277). (B) Images acquired with scanner software version XA30 and acquisition protocol “T1_1mm_standard” (*n* = 138). (C) Images acquired with scanner software version VE11 and acquisition protocol “T1_1mm_standard” (*n* = 70). Darkblue points and lines represent the mean IQR at each time point.

We selected participants who underwent two 1 mm anatomical images taken at different time points (T1, T2), with both time points acquired with the same scanner software version and the same acquisition protocol (mean number of days between T1 and T2 = 281.73 ± 308.38). F‐test on linear regression model showed that IQR at T1 was a robust predictor of IQR at T2 (all *p* < 0.001); *n* = 277 participants with scanner software version VB17 and acquisition protocol “T1_1mm_extended,” F(1, 274) = 57.03, Figure 4A; *n* = 138 participants with scanner software version XA30 and acquisition protocol “T1_1mm_standard” (F(1, 134) = 42.36, Figure 4B); *n* = 70 participants with scanner software version VE11 and acquisition protocol “T1_1mm_standard,” (F(1, 70) = 17.97; Figure 4C).

**FIGURE 5:**
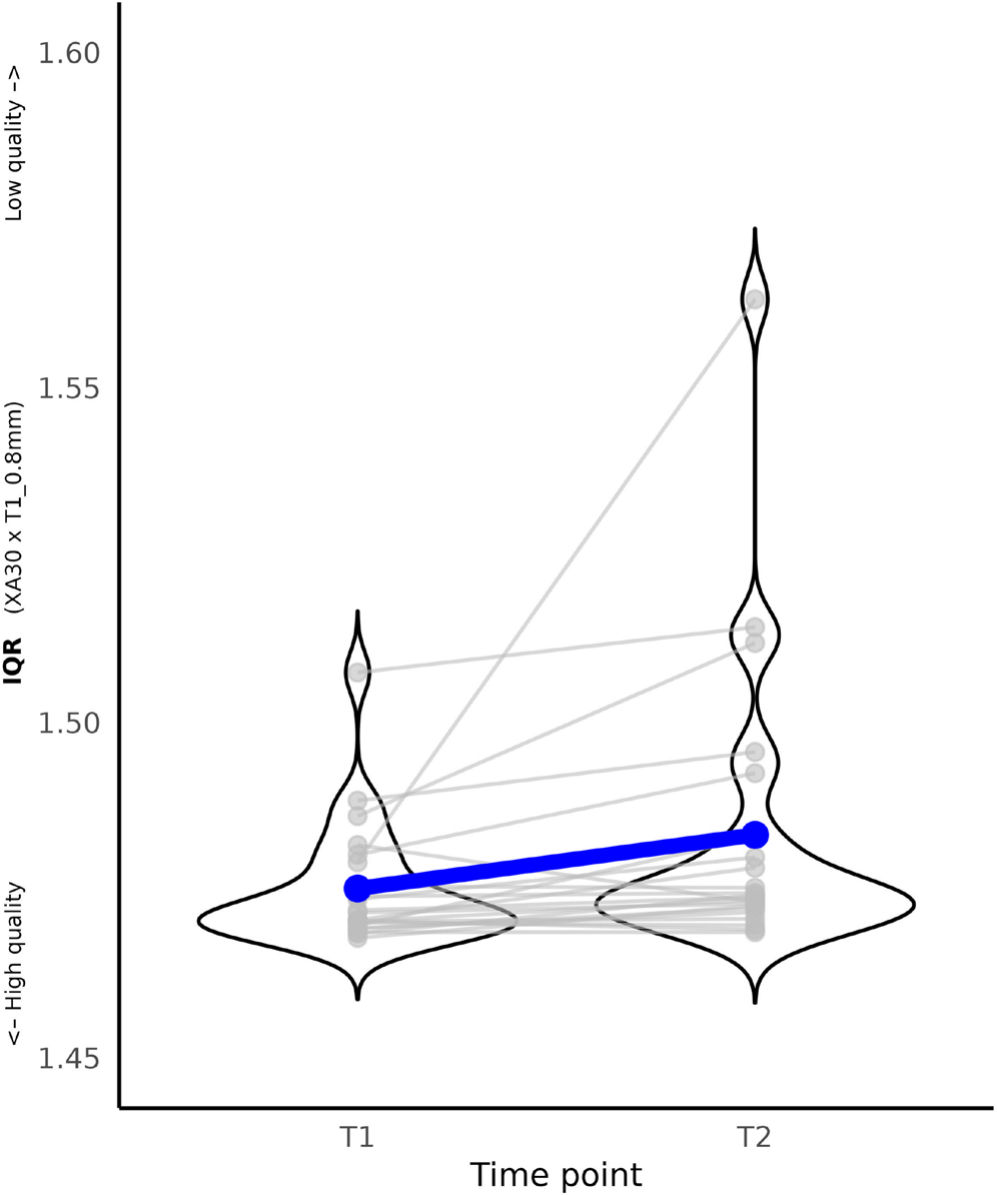
Stability of IQR over time in resolution 0.8 mm (*n* = 22).

We selected participants who underwent two anatomical images taken at different time points (T1, T2) and acquired with 0.8 mm spatial resolution (*n* = 22; mean number of days between T1 and T2 = 162.63 ± 106.73). F‐test on linear regression model showed that IQR at T1 was a robust predictor of IQR at T2 (F(1, 17) = 9.82, *p* = 0.006; Figure 5).

### 3.5 Defacing Effect in 1 mm

**FIGURE 6:**
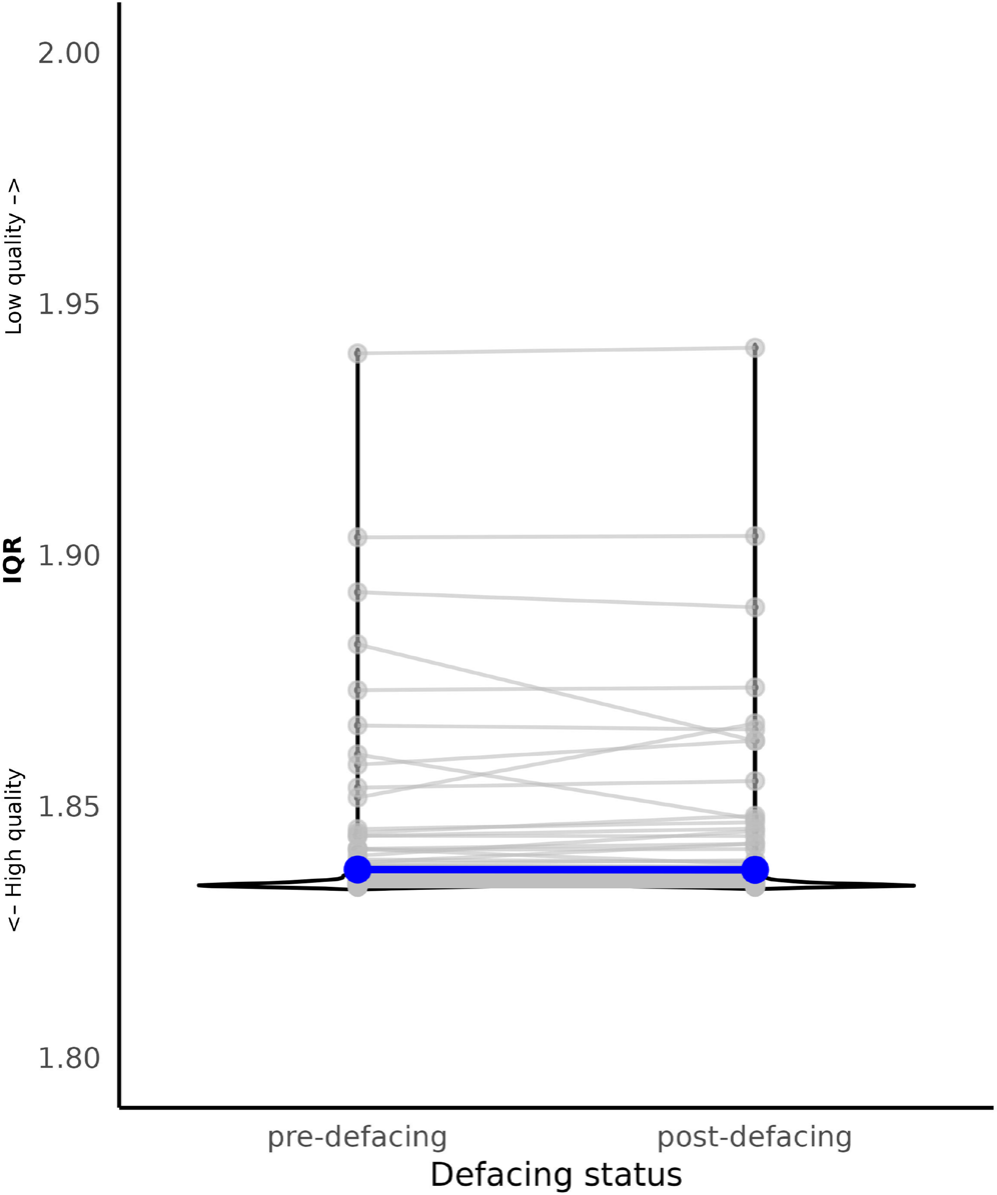
The defacing procedure did not significantly alter IQR (IQR pre‐defacing (left) and IQR post‐defacing (right) (*n* = 190)). Darkblue points and lines represent the mean IQR pre‐ and post‐defacing.

Wilcoxon signed‐rank test showed no significant differences in IQR between pre‐ and post‐defaced images (V = 9734, *p* = 0.383; Figure 6).

### 3.6 Effect of Participant Characteristics

#### 3.6.1 Mental Health Status

**FIGURE 7:**
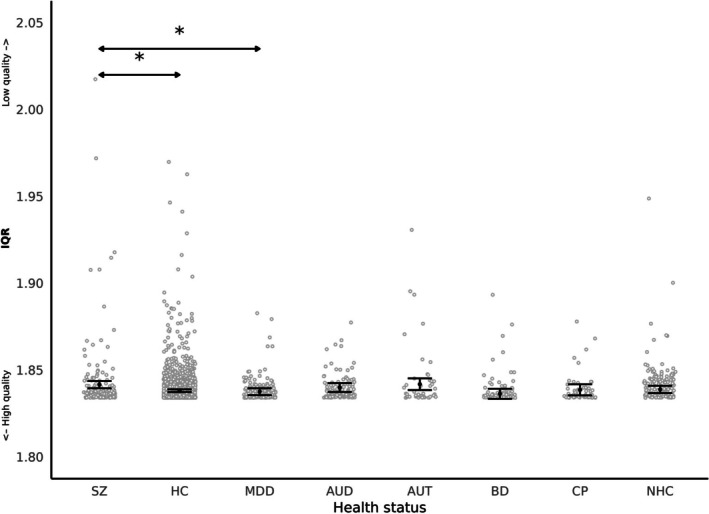
IQR According to health status for images at 1 mm spatial resolution (AUD, Alcohol Use Disorder; AUT, Autism Spectrum Disorder; BD, Bipolar Disorder; CP, Chronic Pain; HC, healthy control; MDD, Major Depressive Disorder; NHC, non‐healthy control with unspecified disease; SZ, Schizophrenia). Each grey point represents an individual data value. Black points and lines indicate the estimated marginal means and confidence intervals for each health status group, averaged over the levels of sex and scanner software version × acquisition protocol combination. T‐test showed significant difference between SZ and HC, SZ and MDD with *p* < 0.05 (*).

In 1 mm spatial resolution, F‐tests revealed significant effects of health status (F(7, 1744) = 2.78, *p* = 0.007). After Tukey correction, two comparisons remained statistically significant (Figure 7): individuals with SZ had a significantly higher IQR than HC (beta = −0.003, 95% CI = [−0.01, 0.000], t(1584) = −3.16, *p* = 0.035), and MDD (beta = −0.004, 95% CI = [−0.01, 0.000], t(1628) = −3.05, *p* = 0.048). Pairwise comparisons between all health status groups are reported in Supporting Information results (Table S1).

In images with a spatial resolution of 0.8 mm, F‐tests revealed non‐significant effect of health status (F(4, 224) = 1.38, *p* = 0.23).

#### 3.6.2 Main Effect of Sex and Age and Their Interaction

In 1 mm resolution, F‐tests showed significant effects of age (F(1, 1615) = 21.37, *p* < 0.001), sex (F(1, 1343) = 16.87, *p* < 0.001) and a significant interaction between age and sex (F(1, 1418) = 3.97, *p* = 0.046). We then examined IQR separately for women and men. T‐tests revealed no significant effect of age on IQR in women (beta = 0, 95% CI = [0, 0.0001], t(910) = 1.28, *p* = 0.202), but a significant effect of age on IQR in men (beta = 0.0002, 95% CI = [0.0001, 0.0003], t(694) = 4.75, *p* < 0.001).

**FIGURE 8:**
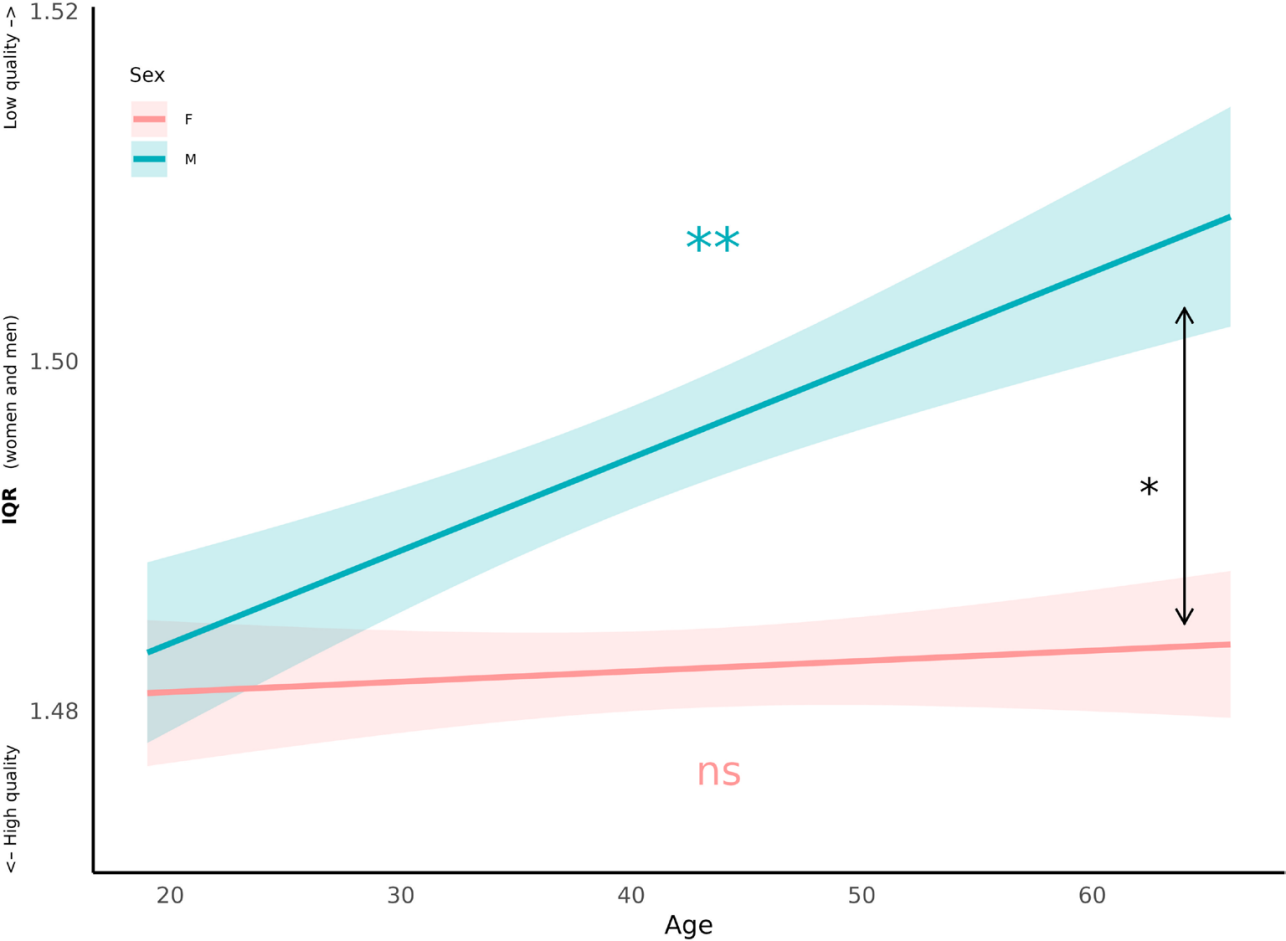
Influence of sex on the relationship between age and IQR in 0.8 mm spatial resolution. Lines represent the linear fit of the data, with the 95% confidence interval indicated by colored areas. F‐test revealed a significant difference between sex with *p* < 0.05 (*). When examining separately for women and men, T‐test revealed a non‐significant effect of age on IQR in women (beta = 0, 95% CI = [−0.0006, 0.0006], t(135) = 0.118, *p* = 0.906; ns) but a significant effect of age on IQR in men (beta = 0.0012, 95% CI = [0.0005, 0.0022], t(87) = 2.65, *p* = 0.009; **).

In 0.8 mm resolution, we found an age‐by‐sex interaction (F(1, 224) = 6.36, *p* = 0.025; Figure 8). F‐tests revealed significant effects of age (F(1, 227) = 6.35, *p* = 0.012) but no main effects of sex (F(1, 225) = 0.88, *p* = 0.347). When we examined IQR separately for women and men, there was no significant effect of age on IQR in women (beta = 0, 95% CI = [−0.0006, 0.0006], t(135) = 0.118, *p* = 0.906), but a significant effect of age on IQR in men (beta = 0.0012, 95% CI = [0.0005, 0.0022], t(87) = 2.65, *p* = 0.009; Figure 8).

### 3.7 Models Performance

For 1 mm resolution images, fixed effects from technical and participant factors explained 5.9% of the IQR variance (marginal R^2^). For 0.8 mm images, participant‐related fixed effects accounted for 10.71% of the variance.

## 4 Discussion

We investigated the impact of various factors—scanner site, coil design, scanner software versions, acquisition protocols including different spatial resolutions, and participant characteristics (e.g., sex, age, and health status)—on MRI quality. While previous studies often examined isolated factors affecting image quality, we present a comprehensive analysis of their combined effects and interactions. Using 2779 T1‐weighted images from 1473 participants (910 healthy, 563 clinical), we provide deeper insights into how these factors jointly impact image quality.

### 4.1 Scanner Site, Hardware, Software

There were no significant differences in IQR between data acquired at two different scanner sites (same manufacturer, hardware and software version). There were also no significant differences in IQR between major scanner updates: that is, between VB17 and VE11 and between VB17 and XA30, which is in line with previous studies (Han et al. 2006; Jovicich et al. 2009). Previous studies, however, reported differences in signal quality related to scanner manufacturer (Siemens, Philips, GE) and magnetic field strength (1.5 vs. 3T) (Han et al. 2006; Jovicich et al. 2009; Kruggel et al. 2009). All our data were collected using 3T Siemens scanners, and this might explain that we did not find significant differences in IQR across sites or major scanner updates.

Noteworthy, IQR was significantly different between “older” software versions (before 2021): VB17 showed higher IQR than VB15 under identical protocols (“T1_1mm_short”) and despite no hardware change. This was unexpected, as software updates typically enhance performance. A previous study using GE scanner also reported significant differences in structural measures after software update (no hardware upgrade) (Takao et al. 2013). Moreover, the authors reported that scanner software update had effects on longitudinal structural metrics comparable to those of using different scanners at different time points (Takao et al. 2013). Some software updates can introduce changes in image reconstruction algorithms, data processing parameters, or signal filtering techniques, potentially affecting the image quality. In the aforementioned study, the authors did not examine quality metrics, it is therefore unclear if the update in software version reported was associated with changes in image quality. This underscores the importance of manufacturers standardizing their calibration processes and algorithms to improve consistency and ensure reliable comparisons across scanners, particularly in multi‐center studies. Further research is needed to confirm these effects across scanners and software versions.

We did not find an effect of the *coil type* on IQR. This finding is not in line with previous studies which reported higher cortical and subcortical SNR for the 64‐compared with the 32‐channel coil. Differences between these outcomes could be related to the acceleration factor GRAPPA (*R* = 4 in previous studies and *R* = 2 in the present data). Indeed, increased central and peripheral SNR for the 64‐ compared with the 32‐channel coil was found to be associated with higher acceleration factors (*R* > 3; Keil et al. 2013; Schmitt and Rieger 2021).

### 4.2 Acquisition Protocol

*Acquisition protocols* had a significant impact on IQR. Namely the protocol “T1_1mm_extended” provided higher IQR, lower quality, compared with “T1_1mm_short” and “T1_1mm_long” protocols. Importantly, these protocols were acquired with the same scanner software version, and therefore the difference can be solely attributed to acquisition parameters. T1‐weighted image contrast depends on a mix of several physical parameters (e.g., TR/TE, bandwidth, number of slices, voxel size…), which are often interdependent. Thus, interpreting the role of individual parameters as we do in the following sections requires caution.

Compared with “T1_1mm_short” and “T1_1mm_long,” the “T1_1mm_extended” acquisition protocol has a lower bandwidth (resp. 150, 170, 130 Hz/px, see Table 2) and a higher number of slices (resp., 176, 176, 192). High bandwidth likely reduces susceptibility effects, improving image SNR and potentially contributing to the superior image quality observed in the “T1_1mm_short” and “T1_1mm_long” compared to the “T1_1mm_extended” protocol. Moreover, while the “T1_1mm_extended” protocol covers more slices, this comes at the cost of relatively longer TR for a given slice, reducing sensitivity and contrast. In contrast, the shorter TR in the “T1_1mm_short” and “T1_1mm_standard” protocols enhances contrast, resulting in higher regional grey matter contrast.

The protocol “standard” provided lower IQR compared with protocol “extended.” These two protocols were however acquired on different scanner software versions (resp. VB17 and both XA30/VE11). Therefore, due to the collinearity between protocols and scanner software, it remains unclear whether the observed differences are primarily related to variations in parameters (e.g., TR) or to the software updates. Notably, for the same acquisition protocols (“T1_1mm_short” and “T1_1mm_long”) there were no significant differences in IQR between VB17, XA30 and VE11 software versions. The difference between “T1_1mm_standard” and “T1_1mm_extended” are the TR (resp. 2000, 2300 ms) and the flip angle (resp. 15, 9**°**). These parameters likely contributed to a higher SNR and improved IQR observed with the “T1_1mm_standard” compared to “T1_1mm_extended.”

### 4.3 Spatial Resolution

Images at high resolution (0.8 mm) have a higher quality compared with 1 mm. The influence of spatial resolution on image quality remains relatively understudied. Nevertheless, higher spatial resolution has demonstrated the ability to provide more accurate cortical measurements, though it is more vulnerable to motion artifacts, likely due to longer scanning times (Ai et al. 2021). A previous study showed that compared with 1 mm, 0.8 mm spatial resolution was associated with more sensitive detection of atrophy patterns within subjects and stronger effect sizes between groups (Fazlollahi et al. 2023). Therefore, these findings highlight the value of using high‐resolution structural images, indicating that their benefits outweigh any potential drawbacks, particularly in studies requiring detailed cortical measurements or examining subtle differences between patient groups.

### 4.4 Longitudinal Assessment of IQR

IQR at T1 significantly predicts IQR at T2 (mean time interval: 162 days). This finding was independent of spatial resolution, scanner software, and protocol and is in line with previous findings (Jovicich et al. 2009; Takao et al. 2013). These findings support the conclusion that IQR is a robust measure with high stability over time.

### 4.5 Defacing

*Defacing* did not significantly alter IQR. The algorithm employed in this study, that is, *mideface*, was designed to be minimally invasive. Previous studies comparing various algorithms (e.g., AFNI, FreeSurfer, spm, FSL, python) did, however, report a significant impact of defacing on structural measures, but not consistently (Bhalerao et al. 2022; Cali et al. 2023; Rubbert et al. 2022). The differences between these defacing algorithms arise from variations in their pipelines for removing facial information (Gao et al. 2023). Some software packages remove extensive portions of facial features (AFNI, SPM, Pydeface), while others adopt a more conservative approach (FSL, mideface). These discrepancies in the extent of facial feature removal can impact downstream analyses, particularly when facial structures near the brain are inadvertently altered, potentially affecting image quality or structural metrics. None of the previously published studies utilized *mideface*, a more recent defacing algorithm. This may explain discrepancies with earlier studies. Further research is needed to improve defacing algorithms that protect privacy while supporting data sharing.

### 4.6 Subjects Characteristics: Age, Sex, and Mental Health Status

IQR was lower in females compared with males, with an interaction between sex and age indicating that IQR increased with age in males but remained stable in females. Importantly, these changes are not due to scanner software or health status, and align with the limited published studies on sex differences in brain aging. Prior research shows faster age‐related declines in brain volume and myelin in men, suggesting that women may be more resilient to aging (Canales‐Rodríguez et al. 2021). The neurobiological and functional bases of these differences remain unclear but may involve biological and environmental influences on brain structure (Liu et al. 2020). Age‐related reductions in brain volume and tissue composition have also been widely reported (Fjell and Walhovd 2010; Yeatman et al. 2014) with potential effects on signal quality (Salat et al. 2009).

IQR was statistically different between SZ patients and HC and MDD, but not in the other clinical groups (AUD, AUT, BD, CP, NHC) after controlling for sex and age. IQR calculates a composite score that reflects basic image properties, noise, and geometric distortions, but does not provide individual values for each specific measure. The higher IQR in SZ was found for images acquired at 1 mm only, and not at 0.8 mm spatial resolution. Despite the 0.8 mm resolution offering finer detail, it may have introduced a lower SNR or partial volume effects, which could reduce the ability to detect meaningful group differences. Moreover, disease differences in structural measures have previously been shown between SZ and healthy controls and have been associated with illness duration (Chand et al. 2020). Further analyses examining relationships between IQR, medication, and clinical symptoms might shed light on the structural differences between clinical conditions.

### 4.7 Limitations and Future Directions

Motion artifacts can significantly affect image quality (Nárai et al. 2022; Savalia et al. 2017) by introducing blurring, ghosting, and intensity inhomogeneities and could potentially influence our study conclusions. To investigate this further, we implemented an additional procedure for motion artifact exclusion. We used two motion‐related metrics from MRIQC (Esteban et al., 2017): the Coefficient of Joint Variation (CJV) and the Entropy Focus Criterion (EFC). Images with CJV or EFC values exceeding three standard deviations above the mean (*n* = 40) were visually inspected by two independent raters (LR and JA). Fourteen images were excluded due to detectable motion artifacts, including even mild cases. All statistical analyses were repeated after these exclusions, and the results remained unchanged. Nonetheless, further work is needed to quantify specific sources of artifact more precisely and to disentangle their individual effects on image quality.

The choice of the quality metric, IQR, a composite measure of image quality obtained from the CAT12 toolbox (Gaser et al. 2024) may influence some of the results reported. Complementary indices such as signal‐to‐noise ratio (SNR), contrast‐to‐noise ratio (CNR), and foreground‐to‐background energy ratio (mean energy of image values) each reflect different aspects of image degradation—such as noise, contrast, and spatial inhomogeneity. Using these metrics in combination could provide a more comprehensive characterization of image quality.

In this study, we focused on IQR as a comprehensive measure of overall image quality. Further research is needed to examine specific structural properties within gray matter (GM), white matter (WM), and across cortical and subcortical regions. This could include evaluating metrics such as cortical thickness, cortical volume, and WM/GM contrast, which provide more detailed insights into localized image quality and tissue differentiation.

We did not account for body height, weight or medication use in our analyses, because these factors were outside the scope of the current study. However, the preprocessing of MRI data involved normalization to intracranial volume (TIV), which has been shown to correlate with body height (Kruggel 2006). This process may have consequently mitigated potential confounding effects of height and weight. A separate study should specifically address participant‐related variables, including BMI, medication use, and clinical group status to explore their potential impact on image quality metrics.

## 5 Conclusion

This study highlights the critical factors influencing image quality, including scanner hardware, software version, acquisition protocols, and participant characteristics, as assessed through the IQR metric. The stability of IQR across repeated scans and its insensitivity to defacing confirms its robustness. Variations in image quality stemming from methodological and participant‐related factors highlight the importance of accounting for individual variability. These findings emphasize the need for standardized practices to ensure consistent image quality, particularly in multi‐center studies, and for further research to explore how these factors interact to affect structural measurements.

## Author Contributions

**Lisa Raoul:** study design, data preprocessing and analysis, interpretation, manuscript writing. **Anastasia Benedyk:** study design, data preprocessing, interpretation, manuscript editing. **Oksana Berhe:** study design, data preprocessing, interpretation, manuscript editing. **Thomas Leon Kremer:** study design, data preprocessing, interpretation, manuscript editing. **Malika Renz:** study design, data preprocessing, interpretation, manuscript editing. **Yuchen Lin:** data preprocessing, interpretation, manuscript editing. **Niharika Roychoudhury:** data preprocessing, interpretation, manuscript editing. **Alexander Moldavski:** data preprocessing, interpretation, manuscript editing. **Ali Ghadami:** data preprocessing, interpretation, manuscript editing. **Abhijit Sreepada:** data preprocessing, interpretation, manuscript editing. **Marvin Ganz:** data preprocessing, interpretation, manuscript editing. **Markus Sack:** feedback, interpretation, manuscript editing. **Matthias Ruf:** feedback, interpretation, manuscript editing. **Robert Becker:** feedback, interpretation, manuscript editing. **Andreas Meyer‐Lindenberg:** study design, interpretation, manuscript editing. **Heike Tost:** study design, interpretation, manuscript editing. **Jamila Andoh:** study design, interpretation, manuscript writing and editing.

## Conflicts of Interest

A.M.‐L. has received consultant fees from the Daimler and Benz Foundation, EPFL Brain Mind Institute, Fondation FondaMental, Hector II Foundation, Invisio, Janssen‐Cilag GmbH, Lundbeck A/S, Lundbeckfonden, Lundbeck Neuroscience Foundation, Neurotorium, MedinCell, The LOOP Zürich, University Medical Center Utrecht, University of Washington, the Mental Wellbeing Association and the von Behring‐Röntgen Foundation; speaker fees from Ärztekammer Nordrhein, Caritas, Clarivate, the German Society for Neuroscientific Assessment, Gentner Verlag, the State Medical Association Baden‐ Württemberg, LWL Bochum, Northwell Health, Ruhr University Bochum, Penn State University, the Society of Biological Psychiatry, the University Prague and Vitos Klinik Rheingau; and editorial or author fees from the American Association for the Advancement of Science, the European College of Neuropsychopharmacology, Servier Int. and Thieme Verlag. U.E.‐P. reports consultancy for Boehringer Ingelheim and speaker honorarium from Angelini Pharma. The other authors declare no competing interests.

## Supporting information


**Data S1.** Supporting Information.

## Data Availability

The data that support the findings of this study are openly available in MRI quality factors at https://osf.io/87ysv/?view_only=b6ee2707bdf943a8b88c8cd2da154919, reference number 10.17605/OSF.IO/87YSV.
