# Supplementary material for: Analysis of Factors Affecting Quality in Structural Magnetic Resonance Images
Source: Hum Brain Mapp. 2025 Aug 1;46(11):e70271. doi: 10.1002/hbm.70271 (PMC12315249; doi:10.1002/hbm.70271)
Supplement: Supplementary file 1 — Data S1. Supporting Information. [file HBM-46-e70271-s001.docx]

# Supplementary Material

## S1 Health status differences in IQR

| Contrasts | Estimated difference | SE | Confidence limits | df | t.ratio | p.value |
| --- | --- | --- | --- | --- | --- | --- |
| **HC - SZ** | **-0.003** | **0.001** | **-0.007, 0.000** | **1584.304** | **-3.157** | **0.035** |
| **MDD - SZ** | **-0.004** | **0.001** | **-0.008, 0.000** | **1628.41** | **-3.051** | **0.048** |
| BD - SZ | -0.005 | 0.002 | -0.011, 0.000 | 1351.427 | -3.002 | 0.055 |
| AUT - BD | 0.006 | 0.002 | -0.001, 0.012 | 1831.292 | 2.519 | 0.188 |
| AUT - MDD | 0.004 | 0.002 | -0.002, 0.010 | 2112.551 | 2.196 | 0.354 |
| HC - AUT | -0.004 | 0.002 | -0.009, 0.001 | 2226.897 | -2.169 | 0.371 |
| NHC - SZ | -0.003 | 0.001 | -0.007, 0.002 | 1536.239 | -1.897 | 0.553 |
| AUD - BD | 0.004 | 0.002 | -0.002, 0.010 | 1601.44 | 1.825 | 0.603 |
| AUD - MDD | 0.002 | 0.001 | -0.002, 0.007 | 1783.986 | 1.686 | 0.696 |
| CP - SZ | -0.003 | 0.002 | -0.008, 0.002 | 2186.556 | -1.662 | 0.712 |
| AUT - NHC | 0.003 | 0.002 | -0.003, 0.009 | 2072.986 | 1.545 | 0.783 |
| BD - NHC | -0.003 | 0.002 | -0.008, 0.003 | 1349.659 | -1.433 | 0.842 |
| AUT - CP | 0.003 | 0.002 | -0.004, 0.010 | 2287.985 | 1.369 | 0.871 |
| HC - AUD | -0.002 | 0.001 | -0.006, 0.002 | 1979.653 | -1.309 | 0.896 |
| HC - BD | 0.002 | 0.001 | -0.003, 0.006 | 1295.04 | 1.198 | 0.933 |
| AUD - SZ | -0.002 | 0.002 | -0.006, 0.003 | 1835.076 | -1.106 | 0.956 |
| BD - CP | -0.002 | 0.002 | -0.009, 0.004 | 1885.965 | -1.053 | 0.966 |
| MDD - NHC | -0.001 | 0.001 | -0.006, 0.003 | 1584.318 | -0.905 | 0.986 |
| AUD - AUT | -0.002 | 0.002 | -0.008, 0.005 | 2169.132 | -0.888 | 0.987 |
| AUD - CP | 0.001 | 0.002 | -0.004, 0.007 | 2092.598 | 0.755 | 0.995 |
| HC - NHC | -0.001 | 0.001 | -0.004, 0.002 | 1379.817 | -0.686 | 0.997 |
| BD - MDD | -0.001 | 0.002 | -0.007, 0.004 | 1376.448 | -0.693 | 0.997 |
| AUD - NHC | 0.001 | 0.002 | -0.004, 0.006 | 1811.261 | 0.644 | 0.998 |
| CP - MDD | 0.001 | 0.002 | -0.004, 0.006 | 2153.997 | 0.632 | 0.998 |
| HC - MDD | 0.001 | 0.001 | -0.003, 0.004 | 1667.145 | 0.532 | 0.999 |
| HC - CP | 0 | 0.002 | -0.006, 0.005 | 2283.232 | -0.292 | 1 |
| AUT - SZ | 0 | 0.002 | -0.006, 0.006 | 2081.355 | 0.124 | 1 |
| CP - NHC | 0 | 0.002 | -0.006, 0.006 | 2105.66 | -0.115 | 1 |

# *S1: Pairwise comparisons on estimated mean according to participant's health status, p values Turkey corrected. Anatomical data were all collected at 1mm spatial resolution.*
